# Rationale and methods of an Evaluation of the Effectiveness of the Community Paramedicine at Home (CP@home) program for frequent users of emergency medical services in multiple Ontario regions: a study protocol for a randomized controlled trial

**DOI:** 10.1186/s13063-018-3107-4

**Published:** 2019-01-23

**Authors:** Gina Agarwal, Melissa Pirrie, Brent McLeod, Ricardo Angeles, Walter Tavares, Francine Marzanek, Lehana Thabane

**Affiliations:** 1 Department of Family Medicine, McMaster University, 100 Main Street W, Hamilton, ON L8P 1H6 Canada; 2 Department of Health Research Methods, Evidence, and Impact, McMaster University, 1280 Main Street West, Hamilton, ON L8S 4L8 Canada; 3 Hamilton Paramedic Services, Hamilton, ON Canada; 4 The Wilson Centre, Department of Medicine, University of Toronto/University Health Network, 200 Elizabeth Street, Toronto, ON M5G 2C4 Canada; 5 Post-MD Education, University of Toronto, Toronto, ON Canada; 6 Paramedic and Senior Services, Community and Health Services Department, Regional Municipality of York, Newmarket, ON Canada; 7 St. Joseph’s Healthcare Hamilton, McMaster University, 25 Charlton Avenue East, Hamilton, ON L8N 1Y2 Canada

**Keywords:** Community paramedicine, Health-care Utilization, emergency medical services, primary care, seniors, complex needs

## Abstract

**Background:**

Frequent users of emergency medical services for issues that could be more appropriately managed through non-urgent care deplete the limited resources of the health-care system. Community paramedicine is an emerging field that extends the role of paramedics beyond the traditional emergency response. The goal of the current study is to evaluate the impact of a community paramedicine home-visit intervention with frequent users on reducing ambulance calls, hospital visits, and admissions. The study will also provide a cross-sectional description of the characteristics of frequent users of emergency medical services.

**Methods/design:**

An open-label, pragmatic, randomized controlled trial with parallel intervention and control groups will be conducted in four paramedic services in Ontario. The sample size has been calculated as 261 per group for a 25% reduction in ambulance calls. Eligible participants will be frequent callers (three or more calls in 6 months), individuals who call for at least one lift assist, or individuals referred to the program by a paramedic. Individuals will be randomly allocated to receive either the Community Paramedicine at Home (CP@home) program intervention or their usual care (control). Intervention participants will receive up to three visits from a community paramedic, who will conduct health risk assessments, provide health promotion and education, provide referrals to local resources, and fax reports back to the family physician. Data will be collected from administrative databases (e.g., paramedic services), a custom CP@home program database, participant surveys, and key informant interviews. An intention-to-treat analysis will be conducted, including descriptive statistics and multi-level modeling to find factors predictive of primary and secondary outcomes. A thematic analysis will be used to analyze the qualitative outcomes. An economic analysis will consider the cost-effectiveness of the program.

**Discussion:**

CP@home has the potential to reduce the health-care system burden significantly by targeting current frequent users of emergency medical services. By targeting this population, CP@home aims to decrease ambulance calls and emergency department visits, reducing health-care costs and improving the quality of life of a vulnerable population. If successful, CP@home will inform the development of community paramedicine policies and the expanding role of paramedics in regions across Canada.

**Trial Registration:**

ClinicalTrials.gov, NCT02835989. Registered on July 14 2016.

**Electronic supplementary material:**

The online version of this article (10.1186/s13063-018-3107-4) contains supplementary material, which is available to authorized users.

## Background

Frequent 911 calls for emergency medical services (EMS) deplete limited health-care resources. A common, preventable EMS call is when an older adult needs a lift assist; that is, when the individual falls and is unable to pick themselves up due to limited mobility. Some older adults call EMS frequently for lift assists [1–3]. Other preventable calls are from individuals of all ages who repeatedly call EMS for problems that could be handled by more appropriate community and primary care resources, such as for chronic disease management [3]. The average overall cost of the ambulance call and resultant emergency department (ED) visit in Ontario is CAD 1626 [4]. Concurrently, health-care expenditures continue to grow. Therefore, simple interventions that manage common EMS issues related to mobility or pain and reduce risks related to poor mental health, chronic disease, and falls may reduce health-care system burden and provide potential savings.

Community paramedicine (CP) is a promising new extension to paramedicine in which paramedics apply their skills beyond emergency (911) calls to initiate preventative and rehabilitative health and social programs as part of an integrated health-care effort [4–6]. The broad goals of the Community Paramedicine at Home (CP@home) intervention are to decrease complications due to falls, chronic disease, and poor mental health and to improve mobility, quality of life, and health outcomes in vulnerable populations. In doing so, related and more specific goals are also to decrease calls to EMS and ED visits. It is expected that these changes will ultimately lead to more efficient use of health-care resources, improve access to health care, and improve health outcomes among frequent users of EMS.

### Frequent EMS users

Frequent EMS users can be defined as having made four or more calls within 1 year [7–9], though definitions range from three to ten times per year [9–13]. This population falls primarily into two categories: (1) older adults and (2) individuals of all ages with complex health-care needs.

Studies have shown that older adults, those 65 years and older, account for more than a third of all EMS calls related to cardiopulmonary conditions, diabetes, and falls [14–17]. The data indicate that the most frequent users of EMS, hospital transports, and hospital admissions were residents over 65 years old [18]. Recent reports from the Institute for Clinical Evaluative Sciences identified that the level of health-care services used by older adults is driven mainly by the number of chronic conditions they have [19, 20]. Older adults with high comorbidity (>3 chronic conditions) report poorer health, take more prescription medications, and have the highest rate of health-care visits [21]. Consequently, the Institute for Clinical Evaluative Sciences recommends that health-care providers need to work actively with older adults to prevent new chronic conditions and manage existing conditions to avoid complications [19]. In addition, the risk of falls in this population is substantial. It is estimated that one in three persons over the age of 65 is likely to fall at least once each year [22, 23]. Studies in the United States and the United Kingdom suggest that approximately 50% of individuals who call 911 for a lift assist (i.e., have had a fall) will call 911 again within 1 month [1, 2]. Therefore, reducing the risk of falls could have a substantial impact on the health-care system.

Research also indicates that older adults without family support networks have a higher utilization of hospital ED services [24]. An increasing number of older adults are at risk of being socially isolated [25]. This can be due to living alone, the death of family members or friends, retirement, and often, declining health. The World Health Organization reports that social isolation is related to “increased rates of premature death, lower general well-being, more depression, and a higher level of disability from chronic diseases” [26]. With a growing population of individuals over the age of 65 in Canada [27], it is expected that this demand for ED services will increase.

The second category of frequent users of EMS are those with complex needs, who are often further categorized into sub-groups defined by chronic illnesses and mental health issues, including substance abuse [9]. These are preventable and often non-emergent issues that could be handled by primary care. A systematic review by Scott and colleagues in 2014 [9] found no studies on the characteristics of frequent EMS users in Canada, a gap in the available literature. However, a 2015 study in the United Kingdom, which has a universal health-care system like that in Canada, found that frequent callers are a heterogenous group with complex needs [3]. Of the frequent callers, 55% were females and the mean age was 57.6 years, but with a large range (15–98 years) and standard deviation (21.4 years). The most common caller characteristics were frequent medical or clinical needs (64%), mental illness (40%), elderly (38%), unmet social or personal care needs (25%), substance abuse (24%), frequent faller (23%), and high anxiety (11%). The majority of callers had two or more of these characteristics. In this population, 83% required more than one intervention by a case manager and 30% required three or more interventions [3]. In this study, through appropriate interventions, they were able to reduce the median number of EMS calls per individual from five per month to zero per month [3]. This suggests that by implementing interventions linking frequent callers with appropriate preventative and rehabilitative resources in primary care, there may be a beneficial impact on health and health resource utilization.

### An innovative and novel approach to facilitating appropriate health-care use

In 2012, a report by the leadership of Ontario’s Seniors Strategy highlighted the need to deliver innovative, community-based care with the dual goals of enabling older adults to live safely in their own homes and alleviating related pressures on more costly care settings such as acute care hospitals and long-term care services [28]. Current approaches being explored by governments include looking for programs that reallocate existing resources to decrease the unnecessary use of ED resources. The 2012 report recommended a team-based approach, including exploring the expansion of CP programs to support primary care access for older adults [28]. A survey of paramedic services in Ontario in 2013 [29] showed that six regional services were planning to provide CP programing in the future, including chronic disease management services and in-home lifestyle and safety evaluations. A systematic review of CP [6] suggested that this field is expanding quickly and there is an urgent need to establish and test models of CP programs [6, 28].

To have the greatest impact on reducing unnecessary health-care utilization, there needs to be a two-pronged approach [30]:

1. a population-based intervention located in areas of high need
2. an individual-focused intervention targeting specific high users identified by paramedic services

#### Population-based intervention

To address the first item, a population-based intervention, the McMaster Community Paramedicine Research Team developed the Community Health Assessment Program through Emergency Medical Services (CHAP-EMS) [31], which began as a pilot study [4], was evaluated as a 1-year randomized controlled trial (RCT) [31, 32], and is now being sustained by paramedic services under the name ‘Community Paramedicine at Clinic’ or CP@clinic. CP@clinic is a primary prevention community health promotion program that is targeted at seniors living in subsidized housing. The CP@clinic program is a free, weekly, one-on-one drop-in session held in a common area of the social housing and is open to all residents. It involves a cardiovascular disease risk assessment, blood pressure monitoring, and risk assessments for diabetes and falls. The results are sent to the participants’ family physicians. Paramedics also deliver tailored health promotion and disease prevention advice, linking residents to local community resources to assist them in changing their health behaviors and lifestyle. The RCT results for this program are showing promise in a number of different areas, including reductions in EMS calls based on participation rate, blood pressure reduction and monitoring, and improved aspects of health-related quality of life such as anxiety, depression, and mobility [32].

#### Individual-focused intervention

The research program of the McMaster CP Research Team is being expanded to a home-visit CP intervention, called CP@home, for individuals who call EMS for a lift assist or who frequently call EMS for other concerns. Briefly, the community paramedics will make home visits to those identified by the paramedic service, conduct risk assessments, refer the individual to appropriate resources to prevent future EMS utilization, and report back to their family physician. Details of this intervention and the RCT are provided in Methods/design.

The use of paramedics is ideal for this intervention as it will deal with individuals who are frail, seriously ill, or potentially unstable, and who may require immediate assistance [3]. Paramedics are an excellent fit to handle these emergent situations and also, with training, knowledgeable about health promotion and preventive medicine. Paramedics are also suited well for conducting risk assessments, since this is part of their basic training.

This home-visit intervention will complement the work already being conducted by CP@clinic and it will build on the team’s contributions to community-based care and established collaborative relationships with paramedic services, primary care, social housing, public health, and other community services. We hypothesize that the intervention will lead to a significant reduction in repeat EMS calls and ED visits from high-use individuals. This anticipated decrease in EMS calls and ED visits may have implications in terms of health-care savings and increased health-care resource capacity. It is also expected that the intervention will improve the health behaviors of participants, which may contribute to their overall health outcomes. If the study demonstrates that CP@home is beneficial in reducing the number of EMS calls and ED visits (or not), this will inform decision makers when developing CP policies and expanding the role of paramedics in other regions. Furthermore, the study data will provide the required information for an expansion of CP@home and future research studies.

### Objectives and research questions

Our primary objective is to evaluate the CP@home intervention to answer the following research questions:

- What is the difference in the number of repeat EMS calls from individuals who are frequent callers or who call for lift assist following CP@home, compared to their own baseline and also compared to a control group (usual care)?
- What is the effect of a CP intervention focused on in-home chronic disease management, community health service connection, and education on EMS usage on the rate of acute ED visits and hospital admissions in the intervention group compared to the control group?

Our secondary objectives are to examine individual health risk factors (e.g., blood pressure, anxiety, and depression) and additional health utilization outcomes (e.g., primary care visits) in this population. The intervention will also provide a baseline, cross-sectional description of the characteristics of individuals who repeatedly call EMS in Canada, which has been identified as a gap in the current literature [9]. We will also assess the cost-effectiveness of the program by conducting an economic analysis. Finally, we will assess process outcomes (e.g., number of referrals and participant satisfaction) using qualitative and quantitative methods. This report presents Version 3 (3 May 2018) of the study protocol. We followed the Standard Protocol Items Recommendations for Interventional Trials (SPIRIT) Statement 2013 (Additional file 1).

## Methods/design

### Study design

A mixed-methods study design will be used to answer our research questions. An RCT will be conducted in which all community residents meeting the study criteria will be allocated to receive either the CP@home intervention or usual care (control). Primary and secondary outcomes will be evaluated using parallel comparisons between the intervention and control groups as well as comparisons within groups before and after the intervention is implemented (i.e., using baseline metrics). Qualitative and quantitative process evaluation will be conducted to assess for efficiency of intervention delivery, participant compliance, participant satisfaction, and ways of improving the intervention. The qualitative and quantitative process evaluation will help to generate information on experiences with the program and how to improve it. This study has been approved by the Hamilton Integrated Research Ethics Board. Any amendments will be submitted to the ethics board for approval and revisions made to the clinicaltrials.gov registry, as appropriate.

### Participants

Participants will reside in one of four Ontario regions participating in the RCT. Each region’s paramedic service will identify eligible participants from the previous month and the list will be updated regularly. To be eligible to participate, individuals must meet at least one of the following conditions:

1. called EMS three or more times in the last 6 months and called at least once within the previous month
2. called EMS for a lift assist within the previous month
3. be directly referred by paramedics (identified through usual practice)

Individuals living in long-term care facilities and individuals currently involved in a paramedic home-visit program or other paramedic-led frequent user intervention will be excluded. Participation in the CP@home intervention will be voluntary and participants can withdraw at any time.

### Allocation of intervention

**Fig. 1:**
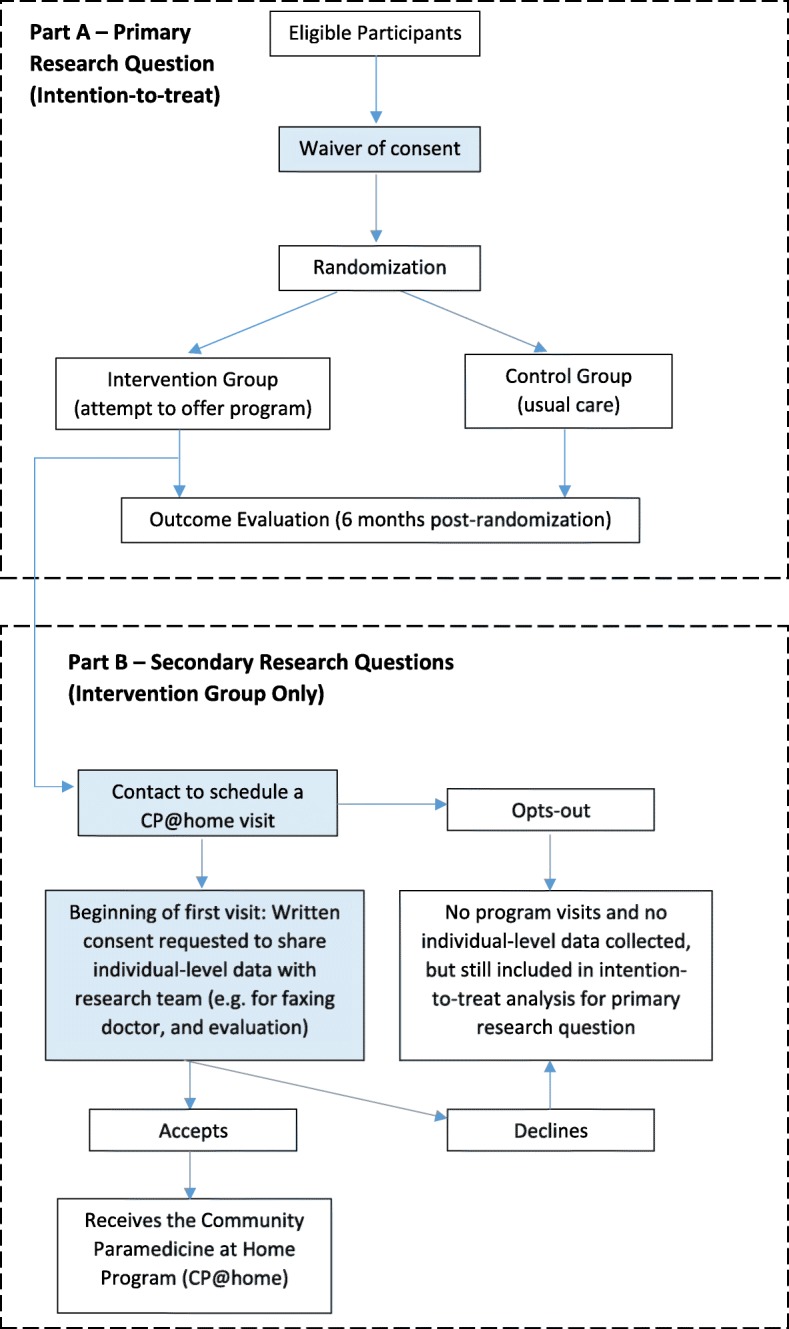
Study flow diagram

Each week, the paramedic service will generate a list of individuals meeting the eligibility criteria. This will be saved to a shared tracking document on a secure virtual network maintained by the McMaster CP Research Team at McMaster University, Hamilton, Ontario. Once the team verify that a participant meets the inclusion and exclusion criteria, they will be randomly allocated to the intervention or control group. The intervention group will receive the CP@home intervention whereas the control group will receive usual care. See Fig. 1 for study flow diagram.

We have secured a waiver of consent for both intervention and control participants prior to randomization since all baseline data collected on participants will be from de-identified secondary data sources. Post-randomization, participants allocated to the intervention group can opt out from receiving the CP@home intervention and no identifiable information will be collected from this group. Those who agree to receive the CP@home intervention will be asked to provide consent since individual information will be collected as part of the intervention and evaluation process.

### Sample size

Our sample size was calculated based on the mean difference in the number of ambulance calls between the intervention and control groups in 6 months. The mean number of ambulance calls per frequent user was 5.3 (standard deviation = 5.7) over a 6-month period for a local paramedic service. Using EMS reductions as our primary outcome for the sample size calculation and the *t*-test as our method of analysis, we will need 261 participants per group to detect a 25% difference between groups assuming standard parameters (alpha = 0.05 and power = 0.8). This difference is considered clinically significant, and a change in one call per high user could result in a resource savings of CAD 1626.00 per participant [4].

### Intervention

**Fig. 2:**
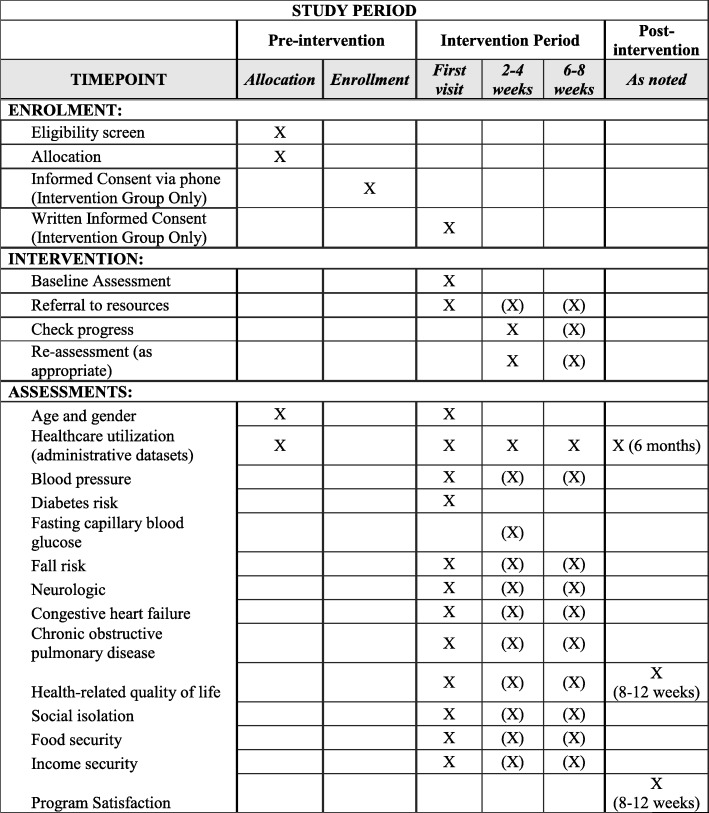
Study enrolment, intervention, and assessments. Items in brackets are completed only if appropriate and are not mandatory for all participants

The intervention group will receive the CP@home program. Community paramedics will conduct a full assessment and risk analysis of participants regarding their overall health status, quality of life, and many social determinants of health. The assessment will include some aspects of CP@clinic (e.g., overall health assessments) but will also include additional evidence-based and previously developed and validated screening assessments (neurologic, cardiac, psychiatric, and social isolation) and connect participants to existing city and community resources. Further program details are provided in Fig. 2. The aim of the CP@home intervention is to provide the participants with the appropriate resources they need based on the assessment findings and the participant’s own perceived needs. It is expected that the ongoing monitoring, identification, and introduction of community resources will improve their health and quality of life, and prevent future crises, thereby decreasing their EMS calls, ED visits, and hospital admissions.

Following consent, the first visit will take approximately 90 min in the participant’s home. Assessments to be completed are:

- Blood pressure and atrial fibrillation using a validated medical device [33]
- Diabetes risk using the validated Canadian Diabetes Risk (CANRISK) tool [34]
  - ○ If necessary, this will be followed up with a fasting capillary blood glucose measurement
- Falls risk using the Timed Up and Go test [35, 36]
- Neurologic assessment using the Mini-Cog Test [37]
- Cardiac assessment for congestive heart failure using the Rise Above Heart Failure tool from the American Heart Association [38]
- Respiratory assessment using the Drive4COPD tool [39]
- Health-related quality of life using the EQ-5D-5 L Tool [40]
  - ○ Sections used will include pain and activities of daily living, such as mobility, self-care, and usual activities.
- Social isolation using the Three-Item Loneliness Scale [41]
- Food security using a brief Hunger Screening tool [42]
- Income security with a tool used by primary care to identify poverty [43]

The assessments will be stored in the CP@home database using an electronic questionnaire in the REDCap platform that collects participant information, calculates risk factors, and summarizes them in a risk factor profile. The CP@home database uses an algorithm to direct participants to the appropriate health-care and community services based on their risk factor profile. If urgent care is required, action will be taken to facilitate transport to an urgent care or emergency facility. Otherwise, based on the risk assessments, referrals will be made to the appropriate resources (e.g., primary care, legal aid clinic, social worker, or local wellness programs) or health education will be provided (e.g., health promotion and appropriate use of EMS). The risk assessment of consenting participants will be sent to their family physician.

A second visit will be made to the participant 2 to 4 weeks following the initial visit for a streamlined follow-up and reassessment, which will last for 30 min. Appointments for succeeding visits are made automatically unless the participant decides not to participate anymore. The duration between appointments will vary based on the availability of participants and whether participants need more time to implement the plan and the advice agreed upon during the previous visit. The schedule for the next visit is agreed at the end of each visit. Further referrals may need to be made at this time, or referrals reinitiated.

A final visit will be made 6 to 8 weeks following the initial visit for a final evaluation of the participant’s situation. It is anticipated that the patient will be discharged from the CP@home intervention at this point. If the patient calls EMS following the third visit and they continue to meet the inclusion criteria, they will be re-entered into the CP@home intervention.

The intervention will be implemented by community paramedics from the local paramedic service who have undergone a structured training program to ensure intervention fidelity. This training encompasses 4 hours of online, interactive training modules, including case studies and observing intervention visits led by a trained paramedic.

### Primary and secondary outcome measures

The primary outcome measure will be the number of repeat EMS calls resulting in the dispatch of an ambulance. The secondary outcomes are the number of ED presentations, the number of hospital admissions in the study populations both 6 months before and after the intervention start date, the number of referrals to community services, and the number of referrals to family physicians. We will also obtain a cross-sectional description of the frequent EMS user population.

### Process evaluation measures

Process measures will assess the intervention implementation in terms of the efficiency of its delivery and compliance, and will include the number of CP@home visits (number of individual visits and number of repeat visits), program delivery (such as completion of various risk assessments), and other participant satisfaction and perception measures (such as ease of implementation, weaknesses, barriers and needed improvements).

### Data gathering procedures

The primary and secondary outcomes will be assessed over two time periods: the 6 months prior to the start of the intervention and the 6 months after the initiation of the intervention. Data will be collected from: (1) the local paramedic service databases (EMS call volumes), (2) the CP@home database, which is completed by the community paramedics (health risk assessments, quality of life, other health-related scales, and community referrals), and (3) administrative databases (ED visits, hospital admissions, and primary care visits). Administrative data will be collected pre- and post-intervention, as well as during the study. Table 1 summarizes the outcomes, data sources, and analyses. Data will be transferred from the paramedic services to the study team through a secure, encrypted virtual private network.

**Table 1:** Summary of outcomes, data sources, and analysis

Outcome	Data source(s)	Data analysis
Primary		
Number of repeat EMS calls resulting in ambulance dispatch in the study population	Paramedic services administrative data	- Comparison between intervention and control groups after the 6-month intervention - Multi-level modeling of factors affecting outcome
Secondary		
Number of ED presentations in the study population	Hospital and ED database Paramedic services administrative data	- Comparison between intervention and control groups after the 6-month intervention - Multi-level modeling of factors affecting outcome
Number of hospital admissions in the study population	Hospital and ED database	
Number of referrals to community services (intervention group only)	CP@home database Paramedic services administrative data	- Descriptive analysis
Number of referrals to family physicians (intervention group only)	CP@home database Paramedic services administrative data	
Tertiary		
Characteristics of frequent users (baseline only)	CP@home database	- Descriptive analysis
Cost-effectiveness of CP@home	CP@home database Paramedic services administrative data	- Economic analysis
Process		
Number of CP@home visits	CP@home database	- Descriptive analysis - Thematic analysis
CP@home participant satisfaction	Self-administered survey Key informant and individual interviews of participants and paramedics	

*ED* emergency department, *EMS* emergency medical services

Physical measures (such as blood pressure), risk assessment scores, and process measures will be collected during the home visits and stored in the CP@home electronic database. Participant satisfaction will be collected through a post-intervention self-administered electronic survey and key informant interviews (KIIs). All participants will be invited to answer the self-administered electronic survey. Six intervention participants and two paramedics implementing the intervention from each site will be invited to participate in the KIIs. The surveys will be conducted during the last CP@home intervention visit. The KIIs will be conducted 6 months after the initiation of the intervention. Participants recently enrolled to the intervention and paramedics who have been implementing the program for at least 1 month in the previous 3 months will be included in the KIIs to maximize the richness of the information and minimize recall bias.

### Data analysis

An intention-to-treat analysis will compare all participants as originally allocated after randomization. This will help to avoid any bias associated with a non-random loss of participants. A sensitivity analysis will compare the results of the intention-to-treat analysis to the per-protocol analysis (which includes only participants who have completed the full program). Outcomes will be analyzed at the individual level and geographic level (e.g., by postal code or ward).

Descriptive statistics will be generated for the baseline demographics. The characteristics of the intervention and control groups will be compared. Correlations statistics will be run between variables. Multi-level modeling will be conducted to find factors predictive of the primary and secondary outcomes. Finally, a thematic analysis of the KIIs and participant feedback surveys will be completed.

Intervention fidelity will be maintained through regular monitoring of the CP@home database (by monitoring referral patterns by EMS personnel and proper use of the screening tools). The main outcomes will be analyzed after completing the participant recruitment process (pre-intervention analysis) and 6 to 9 months after completing the intervention for all participants (post-intervention data and full data analysis).

Both CP@home intervention and health-care resource utilization and costs will be collected for the economic analysis, with a focus on whether the upfront cost of the CP@home intervention is offset by other health-care cost savings [44]. Decision analytic modelling techniques will be used to project final outcomes, like life years and quality adjusted life years, from intermediate outcomes measured during the evaluation period. Our economics team has extensive experience in conducting trial-based and modelling-based evaluations aligned with the needs of health policy makers.

For the quantitative process evaluation, data will be analyzed using frequency and descriptive measures. In the qualitative process evaluation, themes and opinions will be analyzed. A within-case and cross-case analysis will be completed for themes generated to summarize the participants’ and implementers’ experiences with the program.

### Knowledge translation and implications

Involving key stakeholders and community partners (i.e., each region’s paramedic service, Public Health, McMaster University, and the Community Care Access Centre) in a knowledge translation and exchange process will provide opportunities to use the results when making decisions about future health programs, policies, and practices. This group of stakeholders will be involved in developing recommendations for the city council and the dissemination of the results. Dissemination includes the development of an online training tool for community paramedics regarding their expanded role in the community with an emphasis on the health promotion and non-urgent aspect of preventative health care. The educational tool developed by our team could be migrated to the platform used by the Centre for Paramedic Education and Research in Ontario. Paramedicine decision makers can share the new tool and the research results with their provincial and national EMS bodies to promote the CP@home program.

The results of this study will be presented in conferences and published in peer-reviewed and open-access journals to reach broad audiences to enhance research uptake. The potential audience to receive the results from McMaster University, paramedic services, and community partners could include decision makers, policy makers, funding bodies, and knowledge brokers. Ultimately, this program could be used to support a shift in the delivery of primary health care. Sustainability actions will involve working with relevant groups such as health-care professionals, service providers, community health support organizations focusing on older adults (e.g., Community Collaborative of the Local Health Integration Network), and decision makers from the municipal and provincial governments.

Though the setting of this study is Canada, its results could be used by other countries that are promoting CP, including members of the International Roundtable for Community Paramedicine [45]. The high frequency of EMS and ED use is a problem for many developed countries and CP has been advocated as one of the solutions to this problem.

## Discussion

Very little is known about frequent users of EMS, especially in Canada [9], but with increasing demands on the health-care system and limited resources, it is important to study this high-utilization group [3]. The CP role has been developing and expanding in recent years, and a community-based RCT of a wellness clinic model in subsidized housing showed a significant impact on EMS calls [31]. The current study will increase our understanding of the population of frequent EMS users, beyond those in subsidized housing, and the effectiveness of a home-visit CP program in reducing ambulance calls and other health-care utilization (e.g., ED visits and hospital admissions). A similar home-visit program using case managers was effective in the United Kingdom [3].

There are some key challenges to consider when implementing a community-based, pragmatic RCT in a vulnerable population, including intervention fidelity, number of eligible participants, and loss to follow-up. Each of these challenges has been addressed to minimize its potential impact.

### Ensuring that CP@home is properly implemented

Training modules will be developed to ensure that implementers conduct the program properly. Fidelity checks will help in assessing the implementation of the program. Regular quality assurance assessments of the entered data will also be done to monitor the implementation of the program and data collection. To ensure the most accurate results, individuals living in long-term care facilities and individuals currently involved in a paramedic home-visit program or other paramedic-led frequent user intervention will be excluded from the analysis. Existing services and programs in the community during the duration of the CP@home study will be noted and accounted for in the analyses due to the possibility of co-intervention.

### Recruiting participants to the program

Hamilton Paramedic Service data indicate that about 1450 patients in a single year would have been eligible for CP@home, if it had existed. Therefore, we are confident that we could recruit enough people from the Hamilton site alone. There are no challenges anticipated with the other sites. We anticipate that identified potential participants will want to take part, as it will give them an opportunity to have a visit from a community paramedic up to three times, and may help to solve their hitherto unsolved health-related issues.

### Loss to follow-up

Loss to follow-up will be a primary concern for our control group as they are only receiving usual care; however, the primary outcome (ambulance calls) and other measures of health-care utilization will be collected from administrative data and are not dependent on individual contact.

CP@home has the potential to significantly reduce the burden on the health-care system and provide potential savings by targeting current frequent EMS users and individuals who call EMS for a lift assist. By targeting this population, CP@home aims to decrease ambulance calls and ED visits, thus health-care resources will be used more efficiently and access to care for the target populations will be improved. At the same time as reducing health-care costs, CP@home is expected to decrease complications due to falls, chronic disease, and poor mental health and to improve mobility, quality of life, and health outcomes in these vulnerable populations. If successful, CP@home will inform the development of CP policies and the expanding role of paramedics across Ontario and across Canada. This study will also inform the expansion of CP@home as a complement to the CP@clinic program.

### Trial status

Recruitment for this trial began in May 2018.

## Additional file

Additional file 1: SPIRIT 2013 Checklist: Recommended items to address in a clinical trial protocol and related documents. (DOC 126 kb)

## Abbreviations

CAD: Canadian dollars
CANRISK: Canadian Diabetes Risk
CHAP-EMS: Community Health Assessment Program through Emergency Medical Services
CP: Community paramedicine
CP@clinic: Community Paramedicine at Clinic
CP@home: Community Paramedicine at Home
ED: Emergency department
EMS: Emergency medical services
KII: Key informant interviews
RCT: Randomized controlled trial
SPIRIT: Standard Protocol Items Recommendations for Interventional Trials
